# Restriction of GAGE protein expression to subpopulations of cancer cells is independent of genotype and may limit the use of GAGE proteins as targets for cancer immunotherapy

**DOI:** 10.1038/sj.bjc.6603163

**Published:** 2006-06-13

**Authors:** M F Gjerstorff, L E Johansen, O Nielsen, K Kock, H J Ditzel

**Affiliations:** 1 Medical Biotechnology Center, Institute of Medical Biology, University of Southern Denmark, Winsloewparken 25, DK-5000, Odense, Denmark; 2 Department of Clinical Pathology, Odense University Hospital, Winsloewparken 15, DK-5000, Odense, Denmark

**Keywords:** GAGE, immunotherapy, cancer/testis antigen, MAGE-A1, immunohistochemistry, testis

## Abstract

The GAGE cancer testis antigen gene family encodes products that can be recognized by autologous T cells, and GAGE proteins have been suggested as potential targets for cancer immunotherapy. Analysis of GAGE expression in tumours has primarily been performed at the level of gene transcription, whereas little is known about GAGE expression at the protein level. To evaluate the potential of GAGE proteins as targets for cancer-specific immunotherapy, we studied the expression of these proteins in normal and malignant cells/tissues using a novel panel of monoclonal antibodies. Immunohistochemical analysis of more than 250 cancer specimens demonstrated that GAGE proteins were frequently expressed in numerous cancer types and correlated with the expression of the cancer testis antigens MAGE-A1 and NY-ESO-1. Significant intercellular and subcellular differences in GAGE protein levels were observed, and most GAGE-positive tumours also contained cancer cells lacking GAGE expression. Studies of genetically homogenous cell lines with similar intercellular heterogeneous GAGE expression showed that GAGE expression was not associated with a specific genotype, but defined a phenotypically distinct population of cells. Surprisingly, in normal tissues we found that GAGE proteins were not restricted to testis, but were also present in a subset of oocytes of resting primordial follicles and in maturing oocytes. This is the first time that a cancer testis antigen has been reported in postfoetal oocytes. The lack of GAGE expression in a subset of cancer cells within GAGE-positive tumours has decisive implications for the development of GAGE-targeted cancer therapy.

Cancer/testis (CT) antigens are proteins encoded by genes that are normally expressed only in the human germline, but which are also expressed in various tumour types. Restriction of CT antigen expression to immunoprivileged normal tissues (Head and Billingham, 1985; Hutter and Dohr, 1998) and high-frequency expression in different types of cancer make them attractive candidates for cancer-specific immunotherapy (Scanlan *et al*, 2002). A large number of CT antigens can be clustered into families containing multiple homologous members (e.g. GAGE, MAGE, NY-ESO-1), while others exist as nonfamily genes. Most CT antigens are chromosome X-linked genes, and the recently published final assembly of chromosome X indicated the existence of more than 99 CT antigens (Ross *et al*, 2005).

The GAGE gene family consists of at least eight genes encoding proteins of high identity, which can be divided into three groups based on different features (De Backer *et al*, 1999). GAGE-1 is the most unique because of an exclusive C-terminal encoded by an exon that has been interrupted in the other GAGE genes. The remaining GAGE members share more than 98% identity, but can be separated into two groups, GAGE-2, -8 and GAGE-3-7, based on the presence of a potentially phosphorylated tyrosine (Y9) in the latter group (Salomon *et al*, 2003). This tyrosine is also absent in GAGE-1. The induction mechanisms of CT antigen expression in tumours have been investigated. Interestingly, expression of several CT antigens, including GAGE, can be induced by the hypomethylating agent 5-aza-2′-deoxycytidine (Sigalotti *et al*, 2002), and it has been shown that induction of transcription correlates with hypomethylation of CT antigen promoters (Janssen *et al*, 1999; De Smet *et al*, 2004). There also seem to be individual differences in the regulation of the transcription of GAGE genes since the GAGE members are not always co-expressed (Kobayashi *et al*, 2000; Eichmuller *et al*, 2002; Eichmuller *et al*, 2003).

GAGE gene transcripts have been found in numerous types of cancers, most frequently in melanomas (De Backer *et al*, 1999; Eichmuller *et al*, 2002) and lung adenocarcinomas (De Backer *et al*, 1999), in which up to 54% of specimens were found to express GAGE, as well as in gastric cancers (Zambon *et al*, 2001; Kong *et al*, 2004) and hepatocellular carcinomas (Kobayashi *et al*, 2000).

Furthermore, GAGE has been correlated with poor prognosis in stomach cancer, esophageal carcinoma and neuroblastoma (Cheung *et al*, 2000; Zambon *et al*, 2001; Kong *et al*, 2004). The function of GAGE proteins remains largely unknown, although antiapoptotic properties of GAGE-7 have been reported (Cilensek *et al*, 2002). If confirmed, this observation may have significant implications for cancer therapy, since inhibition of apoptotic pathways may render cancer cells resistant to therapy.

Evaluation of the potential of GAGE proteins as targets for cancer-specific immunotherapy requires study of GAGE expression on the protein level. The present study describes the analysis of GAGE protein expression in normal and cancer cells and tissues. The expression pattern of GAGE proteins was further compared to that of MAGE-A1 and NY-ESO-1.

## MATERIALS AND METHODS

### Cell cultures

The human medullary breast cancer cell line BrCa-MZ-01 was a gift from Professor V Möbus, Universitätsklinikum, Ulm, Germany. The BrCa-MZ01-A7, -B2, -B7, -K6 and -K11 cell lines was established from BrCa-MZ01 by three rounds of limited dilution cloning. The malignant melanoma cell line MZ2-MEL was a gift from Olivier De Backer, Ludwig Institute for Cancer Research, Brussels, Belgium, while the MDA-MB-231 (HTB-26) and the CHO-K1 cell lines were purchased from the ATCC (Manassas, VA, USA). All cell lines were grown as monolayers in Dulbecco's modified Eagle's medium (Invitrogen, Taastrup, Denmark), supplemented with 10% FCS, 1% nonessential amino acids, 1% L-glutamine, 1% sodium pyruvate, penicillin (100 U ml^−1^) and streptomycin (100 *μ*g ml^−1^).

### Bacterial expression and purification of GAGE-7-glutathione *S*-transferase

The coding sequence of GAGE-7 was amplified from medullary breast cancer cell line BrCa-MZ01 cDNA using primers 5′-CCG GAA TTC ATG AGT TGG CGA GGA AGA TCG-3′ and 5′-ATA GTT TAG CGG CCG CTC AAC ACT GTG ATT GCT TTT CAC CTT CT-3′. The product was digested with *Eco*R1 and *Not*1 and ligated into predigested vector pGEX-4T-1 (Amersham Pharmacia Biotech, Piscataway, NJ, USA) for expression of glutathione *S*-transferase (GST) fusion proteins. *Escherichia coli* BL21, carrying the GAGE-7-pGEX-4T-1 construct, was grown in SB-media at 37°C. When OD_600_ was approximately 1.0, cultures were induced with 0.2 mM isopropyl-beta-D-thiogalactopyranoside for 2 h at 30°C. Bacteria were pelleted, resuspended in PBS with Complete protease inhibitor (Roche Diagnostics, Penzberg, Germany) and lysed by sonication. GAGE-7-GST was purified with GSTrap (Amersham Pharmacia Biotech) in accordance with the manufacturer's recommendations.

### Production and purification of monoclonal antibodies

Balb/c mice were immunized five times at 2-week intervals with 50 *μ*g of GAGE-7-GST emulsified with TiterMax Gold adjuvant (Stratech Scientific Ltd., Cambridgshi, Sohan, UK). At 3, 2 and 1 days before splenic recovery, 15 *μ*g of GAGE-7-GST in PBS was injected into the dorsal tail vein of experimental mice. Hybridomas of mouse spleen B-cells and Sp2/mIl-6 myeloma cells were produced using the polyethylene glycol method (Kohler and Milstein, 1975). Hybridomas producing anti-GAGE-7mAbs were identified by direct ELISA using MaxiSorp plates (Nalge-Nunc International, Kamstrup, Denmark) coated with GAGE-7-GST or GST. Positive clones were re-cloned three times using serial dilution. Hybridomas were cultivated in Dulbecco's modified Eagle's medium (Invitrogen, Taastrup, Denmark), supplemented with 10% FCS, 1% nonessential amino acids, 1% L-glutamine, 1% sodium pyruvate, penicillin (100 U ml^−1^), streptomycin (100 *μ*g ml^−1^) and 0.05 mM 2-mercaptoethanol and mAbs were purified using Protein G conjugated sepharose.

### Mammalian expression of GAGE

GAGE-1, GAGE-2 and GAGE-7 were amplified from cDNA of MZ2-MEL or BrCa-MZ01 cells using the primers: 5′-CTG GAG CTC GCC ACC ATG TGG CGA GGA AGA TCG ACC TAT CGG-3′ (GAGE-1,-2,-8-sense) or 5′-CTG GAG CTC GCC ACC ATG TGG CGA GGA AGA TCG ACC TAT TAT T-3′ (GAGE-3-7-sense) and 5′-CTT GAT ATC ACA CTG TGA TTG CTT TTC ACC TTC TTC AGG CG-3′ (GAGE-2-7-antisense) or 5′-CTT GAT ATC CTC AAG GTT TCC GTG GGG AAA GA-3′ (GAGE-1-antisense). The product was digested with *Eco*RV and *Sac*1 and ligated into predigested vector pCMV-Tag4A (Stratagene, La Jolla, CA, USA) for expression of proteins with C-terminal FLAG tag. pCMV-Tag4A-GAGE-1, pCMV-Tag4A-GAGE-2, pCMV-Tag4A-GAGE-7 constructs or pCMV-Tag4A were transfected into CHO-K1 cells using the JetPei transfection reagent (Poly Plus Transfection, Illkirch, France) in accordance with the manufacturer's recommendations. At 24 h post-transfection the cells were analysed by Western blotting or immunocytochemistry.

### Quantitative RT–PCR

Relative quantification was performed in triplicate using the standard curve method and SYBR Green PCR Master mix (Applied Biosystems, Foster City, CA, USA) in accordance with the recommendations of the producer. The median relative expression levels were normalized with endogenous glyseraldehyde-3-phosphate dehydrogenase levels. The primers for specific amplification were: GAPDH-sense, 5′-TGC ACC ACC AAC TGC TTA GC-3′, GAPDH-antisense, 5′-GGC ATG GAC TGT GGT CAT GAG-3′, GAGE-1,-2,-8-sense, 5′-5′-GAA GAT CGA CCT ATC GGC-3′, GAGE-3-7-sense, 5′-CGA GGA AGA TCG ACC TAT TAT T-3′, GAGE-1-8-antisense, 5′-GCT GGT TCC ACT TCA TCA CTG-3′. The quantifications were performed twice in their entirety, and the similar relative fold changes confirmed the reproducibility of the methods.

### Western blotting

Sub-confluent monolayers of cells were washed twice in PBS, lysed in 50 mM HEPES (PH 7.0), 500 mM NaCl, 1% NP-40 for 30 min on ice and cleared by centrifugation at 15 000 rpm for 10 min at 4°C. Samples were resolved by 4–20% SDS–PAGE and electroblotted onto a PVDF membrane. The membrane was incubated in PBS, 0.1% Tween-20, and 5% nonfat dry milk powder to block remaining protein binding sites, and then incubated with anti-GAGE mAbs M2, M3 or M4 (1/5000; produced in-house) or with anti-GAGE-7mAb clone 26 (1/5000; BD Biosciences, Franklin Lakes, NJ, USA) followed by horseradish peroxidase conjugated goat anti-mouse IgG (1/100 000) (DakoCytomation Denmark A/S, Glostrup, Denmark). All antibody incubations and washing steps were carried out in PBS, 0.1% Tween-20. The immunoreactive bands were visualized with ECL Western Blot kit (Amersham Biosciences, Hilleroed, Denmark).

### Immunohistochemistry

Sections of tissues were cut, deparaffinized, treated with 1.5% H_2_O_2_ in Tris-buffered saline (pH 7.5) for 10 min to block endogenous peroxidase activity, rinsed in distilled H_2_O, demasked processed for antigen retrieval and washed in TNT buffer (0.1 M Tris, 0.15 M NaCl, 0.05% Tween-20, pH 7.5). A panel of antigen retrieval protocols was initially evaluated including microwave boiling for 15 min in (1) T-EG buffer (10 mM Tris, 0.5 mM EGTA, pH 9.0), (2) 10 mM citate buffer, pH 6.0 or (3) Dako Target retrieval solution (Dako S1699), or proteolytic treatment using (4) 0.05% protease type XIV (pronase E, Sigma, cat. no. P5147) in TBS, pH 7.0 for 15 min at 37°C or (5) 0.4% pepsin (Sigma, cat. no. P7012) in 0.01 M HCl for 20 min at 37°C. The microwave boiling in T-EG buffer for 15 min was found to be the optimal antigen retrieval method for both anti-GAGE-1-8, MAGE-A1 and NY-ESO-1mAbs and was used in the successive experiments. Sections were subsequently incubated with anti-GAGE mAbs M2, M3 or M4 (1/100), anti-GAGE-7mAb (1/2000; Clone 26, BD Biosciences), anti-MAGE-1mAb (1/200; Clone MA454, Lab Vision Corporation, Newmarket Suffolk, UK) or anti-NY-ESO-1mAb (1/25; Clone E978, Zymed Laboratories Inc., San Francisco, CA, USA) diluted in antibody diluent (S2022, DAKO Cytomation, Glostrup, Denmark) for 1 h at room temperature. Sections were washed with TNT and incubated with horseradish peroxidase-conjugated ‘Ready-to-use’ EnVision™+ polymer K4001 (DAKO Cytomation) for 30 min, followed by another wash with TNT. The final reaction product was visualized by incubating with 3,3′-diaminobenzidine (DAB)+ substrate-chromogen for 10 min, followed by washing with H_2_O and counterstaining of sections with Mayers hematoxylin before mounting in AquaTex (Merck Inc., Whitehouse Station, NJ, USA). For each experiment, a sample with either an isotype-matched antibody or no primary antibody was included as control.

### Immunocytochemisty

Cells grown as monolayers were fixed in 4% formaldehyde/PBS for 15 min and permeabilized in 0.25% Triton X100, PBS for 10 min. Cells were incubated with 5% normal goat serum, PBS for 30 min and with anti-GAGE mAbs M2, M3, M4 (1/400) or anti-GAGE-7 mAb clone 26 (1/400) for 90 min in 1% normal goat serum, PBS. After washing, the cells were incubated for 60 min in FITC-conjugated goat anti-mouse IgG (1/300; Jackson ImmunoResearch Laboratories, West Grove, PA, USA), supplemented with 5 *μ*g ml^−1^ propidium iodide or 300 nM DAPI for the last 5 min. Cells were washed again and the slides were mounted with Slow Fade in PBS/glycerol (Molecular Probes, Eugene, OR, USA) and visualized using fluorescence confocal microscopy or fluorescence microscopy.

## RESULTS

### Generation and characterization of GAGE-reactive mAbs

To study GAGE expression at the protein level, a panel of mAbs was generated by immunization of mice with purified GAGE-7-GST fusion protein and selection based on their reactivity in ELISA with GAGE-7-GST, but not with GST alone. From a larger panel, three unique clones, mAbs M2, M3 and M4, as determined by sequence analysis of heavy chains, were selected for further characterization.

**Figure 1:**
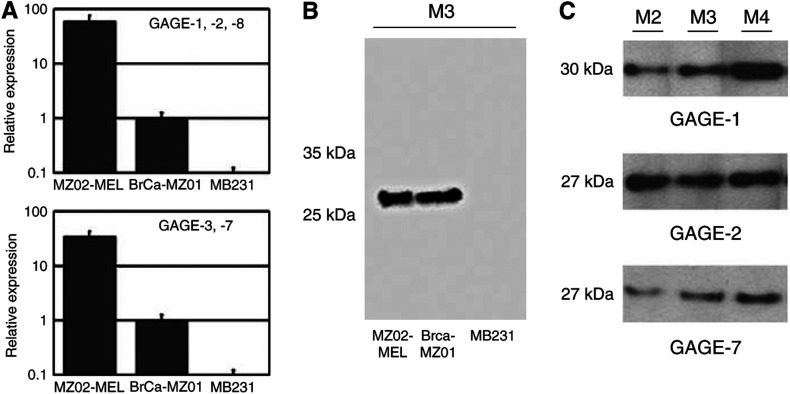
Analysis of the reactivity of anti-GAGE mAbs M2, M3 and M4. (**A**) Both GAGE-1,-2,-8 and GAGE-3-7 were shown, by real-time PCR, to be expressed in melanoma cell line MZ02-MEL and breast cancer cell line BrCa-MZ01, but not in the breast cancer cell line MB231. (**B**) M3 reacted with a 26-kDa protein in lysates of MZ2-MEL and BrCa-MZ01, but not with a lysate of the GAGE-negative cell line MB231. The reactivity of M3 was similar to that of M2 and M4 (not shown). (**C**) mAbs M2, M3 and M4 reacted with a 27-kDa band in lysates of pCMV-Tag4A-GAGE-2 and pCMV-Tag4A-GAGE-7-transfected CHO-K1 cells, corresponding to the size of recombinant GAGE-2 or -7, including the 1-kDa FLAG tag. M2, M3 and M4 also reacted with a 30-kDa band in lysates of pCMV-Tag4A-GAGE-1-transfected CHO-K1 cells, corresponding to the expected size of recombinant GAGE-1, including the 1-kDa FLAG tag.

Two GAGE mRNA-positive cell lines, melanoma cell line MZ2-MEL and breast cancer cell line BrCa-MZ01, and one GAGE mRNA-negative cell line, breast cancer cell line MB231, were identified by quantitative RT–PCR and analysed in Western blots using the purified mAbs (Figure 1A and B). The three mAbs were shown to recognize a 26-kDa band in both reduced and unreduced lysates of MZ2-MEL and BrCa-MZ01, but did not react with a lysate of MB231. MAbs M2, M3 and M4 were also tested for their reactivity with recombinant proteins of each GAGE subgroup, and were shown to recognize bands of approximately 27 kDa in lysates of GAGE-2- and -7-transfected cells, corresponding to the expected size of recombinant GAGE-2 and -7, including the FLAG tag (Figure 1C). Furthermore, all three antibodies recognized a band of approximately 30 kDa in GAGE-1-transfected cells, corresponding to the expected size of GAGE-1. None of the antibodies reacted with lysates of pCMV-Tag4A-transfected cells (negative control). Immunocytochemical analysis confirmed that mAbs M2, M3 and M4 reacted with members of all GAGE subgroups (data not shown).

### Analysis of GAGE protein expression in normal human tissues

**Figure 2:**
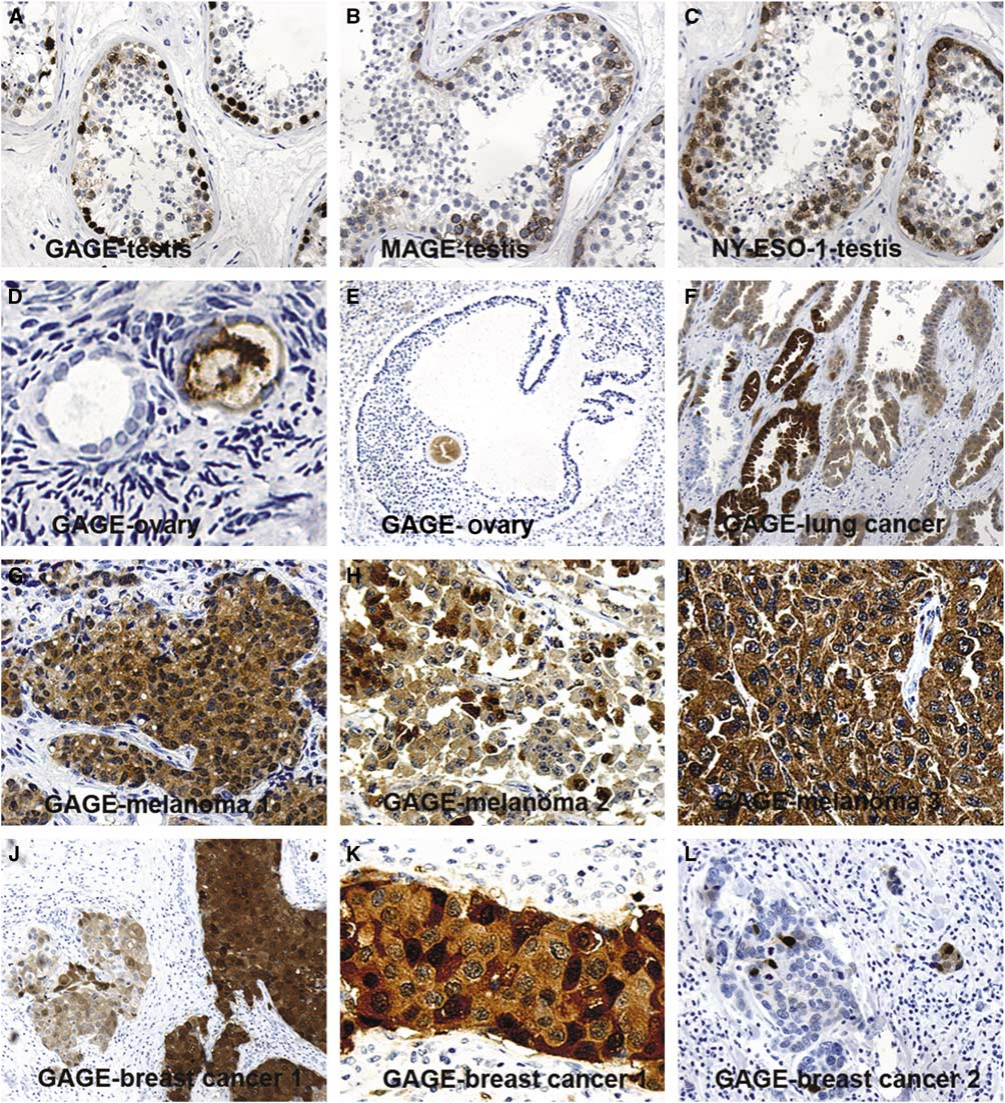
Comparison of GAGE, MAGE-1 and NY-ESO-1 staining in normal testis, and analysis of GAGE protein expression in ovary and different types of cancer. GAGE (**A**), MAGE (**B**) and NY-ESO-1 (**C**) were detected in spermatogonia and primary spermatocytes of the seminiferous tubuli. However, while MAGE-A1 and NY-ESO-1 were located only in the cytoplasm of these cells, GAGE staining was also present, and more intense, in the nuclei. GAGE was also expressed in oocytes of resting (**D**) and maturing (**E**) follicles of normal ovaries, which also contained GAGE-negative oocytes. In three malignant melanomas, all cells exhibited cytoplasmic staining (**G**–**I**), whereas nuclear staining was observed in only two melanomas (**G** and **H**). Heterogeneous staining was also seen in other types of cancer, including lung carcinoma (**F**) and breast carcinoma (**J**–**L**). One breast carcinoma exhibited variations in both cytoplasmic and nuclear GAGE staining among cells (**J** and **K**), while only few cells of another breast cancer specimen were positive (**L**). Magnification: × 10 (**A**, **B**, **C**, **E**, **F**, **J**, **L**), × 20 (**G**–**I**), × 40 (**D**, **K**).

To analyse GAGE-1-8 protein expression in normal tissues, the anti-GAGE mAbs were used for immunohistochemical staining of 24 different normal tissues (Table 1). As expected, testis was positive for GAGE (Figure 2A), and the testicular reactivity was localized to the seminiferous tubuli, where nuclear and cytoplasmic staining of both spermatogonia and primary spermatocytes was observed. All spermatogonia and primary spermatocytes exhibited weak cytoplasmic staining, while strong nuclear staining was seen in spermatogonia and in some primary spermatocytes. Some heterogeneity in the staining intensity of spermatogonia was observed. No staining of secondary spermatocytes, spermatids, Sertoli or Leydig cells was seen.

Interestingly, cytoplasmic GAGE staining was also found in a subset of oocytes (about 30%) of primordial resting follicles in ovary specimens (Figure 2D). No morphological differences between GAGE-positive and GAGE-negative oocytes were detected. A maturing follicle was also identified and the oocyte residing in the cumulus ooforus was intensely stained for GAGE (Figure 2E). No GAGE expression was detected in the other normal tissues examined, as outlined in Table 1, and no apparent differences in the reactivity of mAbs M2, M3 and M4 were observed.

For comparison, the 24 normal tissues were also examined for MAGE-A1 and NY-ESO-1 expression using the mAbs MA454 and E978, respectively (Jungbluth *et al*, 2000; Vaughan *et al*, 2004) (Table 1). Both mAbs reacted with the cytoplasm of spermatogonia and primary spermatocytes, but MAGE-A1 staining was strong in spermatocytes and weaker in spermatogonia, whereas the opposite was observed for NY-ESO-1 (Figure 2B and C). No staining of secondary spermatocytes, spermatids, Sertoli or Leydig cells was seen. In contrast to the anti-GAGE mAbs, neither of the MAGE-A1 the NY-ESO-1 mAbs reacted with the nucleus of spermatogonia and primary spermatocytes, nor did they react with oocytes of the ovary. The staining patterns of MAGE-A1 and NY-ESO-1 antibodies was in accordance with previous studies (Jungbluth *et al*, 2000; Jungbluth *et al*, 2001).

### Analysis of GAGE protein expression in tumours

The GAGE-1-8 specific mAbs were also tested for reactivity with more than 250 cancer specimens, as summarized in Table 2. Among breast carcinomas, lung carcinomas and malignant melanomas, which represented the largest groups in the tumour panel, the incidences of GAGE expression were 12% (5/43), 16% (10/64), and 17% (4/24), respectively (Figure 2F–L). GAGE expression could not be correlated with any specific subtype of these cancers. GAGE-positive specimens were also identified within the smaller panels of bladder carcinomas, liver carcinomas, mesotheliomas, thyroid carcinomas and germinal cell tumours.

The staining pattern within a given tumour varied significantly both in the frequency of tumour cells exhibiting GAGE expression, in the intensity of staining and in the subcellular localization of the staining (Figure 2F–L). For example, in malignant melanomas, most cancer cells were GAGE-positive, while in other cancer types only few cells expressed GAGE. Furthermore, all GAGE-positive melanoma cells exhibited cytoplasmic staining, but there were clear differences in the nuclear staining. Some melanomas showed intense staining of all nuclei (Figure 2G), while other specimens contained only about 50% of positive nuclei (Figure 2H) or no nuclear staining (Figure 2I). In one breast carcinoma (Figure 2L) and one seminoma, very few cells were positive, and these cells were situated in the tumour in a nonclonogenic manner. In another breast carcinoma, focal parts of the tumour were positive (Figure 2J and K). Importantly, cancer cells that did not exhibit GAGE staining were observed in most GAGE-positive tumours.

For comparison, the same panel of 256 tumour specimens was tested for MAGE-A1 and NY-ESO-1 protein expression. MAGE-A1 was detected in the same types of cancer as GAGE, but also in specimens of endometrial carcinoma and pheochromocytoma. Within the large groups of the tumour panel, the highest incidences of MAGE-A1 expression were observed in bladder carcinoma (40%), lung carcinoma (32%), malignant melanoma (21%) and breast carcinoma (11%). NY-ESO-1 expression, as determined by mAb E978 staining, was restricted to a smaller number of cancer types than GAGE and MAGE-A1. Staining was observed in specimens of malignant melanoma, lung carcinoma, lymphoma and germinal cell tumours. Similar to GAGE, NY-ESO-1 was present in both the cytoplasm and nucleus of cancer cells. In contrast, MAGE expression was strictly cytoplasmic in both germ cells and cancer cells (data not shown).

**Figure 3:**
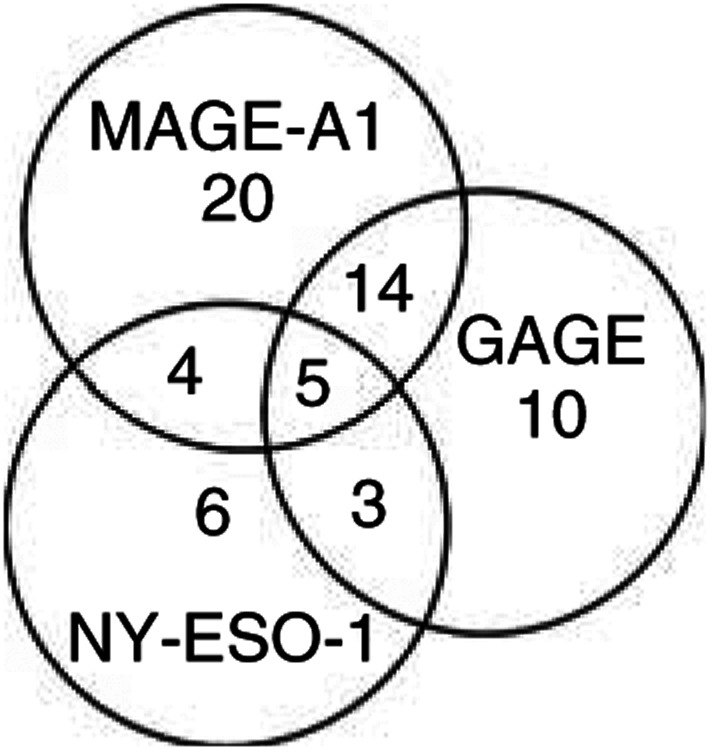
Co-expression of CT antigens in cancers. The numbers indicate tumours that exhibited GAGE, MAGE-A1 and/or NY-ESO-1 staining among the 256 tumours tested. A significant correlation between the expression of GAGE, MAGE-A1 and NY-ESO-1 was observed.

In total, 62/256 (24%) of the tumour specimens included in this analysis were positive for at least one of three CT antigens. In all, 32 (13%) stained for GAGE, 43 (17%) stained for MAGE-A1 and 18 (7%) stained for NY-ESO-1 (Figure 3). Of the 62 CT antigen-positive tumours, 22 (35%) were positive for two CT antigens (most often GAGE and MAGE-A1) and five (8%) were positive for all three CT antigens, suggesting a significant correlation of the expression of these CT antigens. The highest incidence of co-expression was observed in malignant melanoma, where 3/12 CT antigen-positives specimens were stained for both GAGE, MAGE-A1 and NY-ESO-1. Interestingly, within tumours that co-expressed CT antigens, cells that expressed one CT antigen but not another were identified.

### Immunofluorescent microscopy analysis of the subcellular localization of GAGE proteins

**Figure 4:**
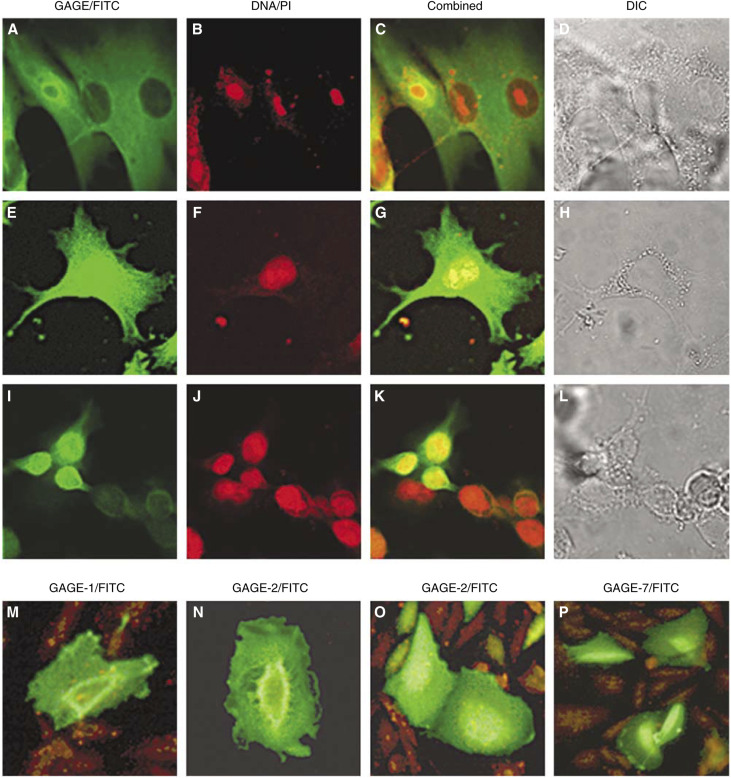
Subcellular localization of GAGE proteins in cancer cell lines. MAb M4 reacted with GAGE in melanoma MZ2-MEL cells (**A**–**H**), exhibiting weak cytoplasmic staining of all cells and strong (**E**–**H**) or no (**A**–**D**) staining of nuclei. A similar staining pattern was observed for breast cancer cell line BrCa-MZ01-A7 (**I**–**L**), but only about 5% of the cells were positively stained. MAb M4 also reacted with recombinant GAGE expressed in CHO-K1 cells (**M**–**P**). The staining patterns of cells transfected with GAGE-1, -2 or -7 constructs were similar to those of MZ2-MEL and BrCa-MZ01-A7 cells. Transfected cells exhibited cytoplasmic staining and strong or no nuclear staining, and some cells had a distinct staining of the nuclear envelope (**M** and **N**), which was also observed in some MZ2-MEL and BrCa-MZ01-A7. Staining was visualized by immunofluorescent confocal microscopy (**A**–**L**) or immunofluorescent microscopy (**M**–**P**). Magnification: × 100.

The subcellular distribution of GAGE proteins was further examined in cells of the melanoma cell line MZ2-MEL and the breast cancer cell line BrCa-MZ01-A7 by confocal microscopy (Figure 4A–L). GAGE proteins were evenly distributed in the cytoplasm of all MZ2-MEL cells, while staining of the nuclei was either intense (Figure 4E–H) or completely absent (Figure 4A–D). In BrCa-MZ01-A7, only about 5% of the cells were stained. As in the MZ2-MEL cells, all the GAGE-positive BrCa-MZ01-A7 cells exhibited cytoplasmic staining, while the nuclei were either intensively stained or negative (Figure 4I–L). In a small number of cells of both cell types, GAGE staining was confined to the nuclear envelope (data not shown). Nucleoli appeared to be negative in all GAGE-positive nuclei.

The subcellular localization of the individual GAGE members was addressed by immunofluorescent microscopy analysis of GAGE-1-, -2- or -7-transfected CHO-K1 cells (Figure 4M–P). The staining patterns of all three GAGE proteins were similar to those of MZ2-MEL and BrCa-MZ01-A7. Transfected cells exhibited cytoplasmic staining and varying nuclear staining, while a minority of cells showed strong staining of the nuclear envelope (Figure 4M and N). No apparent differences in the subcellular localization of GAGE-1, -2 or -7 were observed.

### Heterogeneity of GAGE expression in BrCa-MZ01 subclones

**Figure 5:**
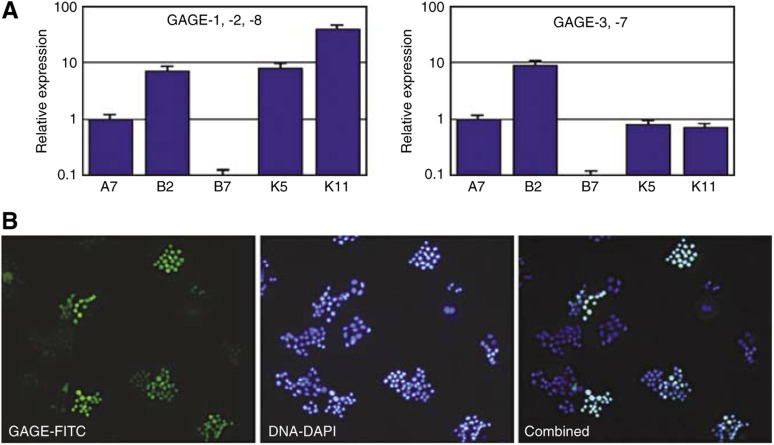
Clonal GAGE expression in BrCa-MZ01 breast cancer cells. (**A**) Subclones of the BrCa-MZ01 cell line were isolated as tested for GAGE expression by real-time PCR. A7, B2, K5 and K11 were all positive for both GAGE-1,-2,-8 and GAGE-3-7, while B7 was completely negative. (**B**) BrCa-MZ01-K11 cells were seeded at low density, allowed to grow for 6 days, and then stained for GAGE (FITC, green, top and bottom panel) and DNA (DAPI, blue, middle and bottom panel). GAGE was clonally expressed in approximately 30% of the genetically-homogeneous BrCa-MZ01-K11 cells, indicating that GAGE expression is independent of genotype. Magnification: × 20.

To determine whether variations in GAGE expression were associated with genetic variability, five cell lines, that is BrCa-MZ01-A7, -B2, -B7, -K6 and -K11, were established from single cells of the original BrCa-MZ01 cell line by three rounds of limited dilution cloning and GAGE expression was assessed. Quantitative PCR showed that cell lines BrCa-MZ01-A7, -B2, -K6 and -K11 expressed GAGE-1,-2,-8 and GAGE-3-7 mRNA, while BrCa-MZ01-B7 was negative for both (Figure 5A). Immunocytochemical analysis further showed that only 5–30% of BrCa-MZ01-A7, -B2, -K6 and -K11 cells were GAGE-positive, and that the positive cells were clonally derived (Figure 5B), suggesting that GAGE expression is not associated with a specific genotype, but with a phenotypically distinct population of cells.

### Comparison of the reactivity of the anti-GAGE mAbs with that of a commercial anti-GAGE-7 antibody

During the latter part of this study, a commercial anti-GAGE-7mAb (clone 26; BD Biosciences) became available. This antibody, which recognized a 26-kDa band, similar to our anti-GAGE mAbs, was recommended only for Western blot applications by the manufacturer, but two recent studies have shown that this mAb is also suitable for immunohistochemical analysis (Luftl *et al*, 2004; Akcakanat *et al*, 2006). To compare the reactivity of this antibody with the reactivity of mAbs M2, M3 and M4, the anti-GAGE-7mAb was tested in parallel with M3 for reactivity with the panel of normal and cancer tissues by immunohistochemistry. The reactivity pattern for the two antibodies was identical within both normal tissues, including reactivity with spermatogonia, primary spermatocytes and oocytes, and within the cancer tissues.

## DISCUSSION

CT antigens are currently a major focus of cancer research due to their potential as targets for cancer-specific immunotherapy. For this purpose, it is essential to determine the types of cancer that express CT antigens, at what frequency, and the pattern of expression within a given tumour, including percentage of positive cells, expression levels and the subcellular localization of expression. The expression of GAGE proteins in tumours has been addressed by RT–PCR in numerous studies, but until recently no anti-GAGE antibodies were available (Luftl *et al*, 2004; Akcakanat *et al*, 2006). In this study, we have generated a panel of GAGE-reactive mAbs and used these to characterize the extent of heterogeneity in GAGE expression in malignancies.

Using our mAbs, GAGE protein expression was identified in specimens of malignant melanoma, breast carcinoma, bladder carcinoma, lung carcinoma, liver carcinoma, thyroid carcinoma, mesothelioma and germinal cell cancers. Importantly, our study demonstrated significant variations in the level of GAGE expression in the different tumours, and within the majority of the tumours analysed we also observed an intercellular heterogeneity of GAGE protein expression that included GAGE-negative cells. This may have important implications for the development of GAGE-targeted cancer vaccines, since GAGE-negative cells within GAGE-positive tumours may escape treatment.

The degree of co-expression of GAGE, MAGE-A1 and NY-ESO-1 genes in tumours was evaluated by immunohistochemical analysis of parallel tissue sections and a significant correlation between the expression of these CT antigens, GAGE and MAGE-A1, in particular, was found among cancer types and specimens. Interestingly, the proteins were not consistently co-expressed and within a tumour that expressed more than one CT antigen subpopulations of cancer cells were observed that were positive for one CT antigen and negative for another.

The frequencies of GAGE-positive tumours within the different cancer types assessed in our immunohistochemical analyses were somewhat lower than the frequencies of previous mRNA-based studies on the same types of cancer. For example, GAGE expression in malignant melanomas was observed in 17% of specimens in our study, while a previous study showed that 30% of malignant melanomas were GAGE-1-8 mRNA-positive (Eichmuller *et al*, 2002). Similar differences were observed for lung carcinoma (16 *vs* 50%), thyroid carcinoma (10 *vs* 30%) and ovarian carcinoma (0 *vs* 30%) (Russo *et al*, 1996; De Backer *et al*, 1999; Ruschenburg *et al*, 1999; Kobayashi *et al*, 2000). Lower frequencies of tumours exhibiting MAGE-A1 and NY-ESO-1 staining, compared to previous mRNA-based studies, were also observed (Li *et al*, 1996; Zambon *et al*, 2001; Kong *et al*, 2004; Wang *et al*, 2004; Li *et al*, 2005). In accordance with our results, a lower frequency of immunohistochemical-positive tumours compared to mRNA-positive tumours was recently reported in a study addressing GAGE, MAGE-A1 and NY-ESO-1 expression in oesophageal carcinomas (Akcakanat *et al*, 2005). These discrepancies are most likely the result of differences in the amount of tumour tissue analysed by the two techniques. When isolating RNA for RT–PCR analysis, a 5 mm cubic tumour block is generally used. In contrast, only a 10 *μ*m section, about 500 times less tissue, is used for immunohistochemistry. As some tumours contain very few CT antigen-positive cells or exhibit CT antigen expression only in focal parts of the tumour, it is more likely that the higher number of cells used in RT–PCR analysis *vs* array-based immunohistochemical analysis will include CT antigen-positive cells. Supporting this, some tumours, which were initially identified as GAGE-negative by immunohistochemistry, were found to contain some GAGE-positive cells, when re-examined using sections obtained from deeper parts of the same tumour blocks. Another, less likely, explanation may be that the sensitivity of immunohistochemical analysis is lower than that of RT–PCR analysis.

Analysis of the subcellular expression of GAGE expression demonstrated that all positive cells exhibited weak cytoplasmic staining and variable nuclear staining in both cancer and normal cells (e.g. germ cells). This suggests that CT antigens are expressed in a natural context when expressed in cancer cells, and thus may play a functional role in these cells. It also supports the hypothesis that CT antigens are expressed as a part of a coordinated gametogenic program that can be activated in cancer cells and that could account for the many similarities between germ cells and cancer cells (Scanlan *et al*, 2002).

To investigate the mechanisms that control the GAGE expression, we also addressed GAGE expression in cancer cell lines. A set of genetically-homogenous subclones were established from the BrCa-MZ01 cell line by three rounds of subcloning. Interestingly, we found that only 5–30% of the cells of these subclones expressed GAGE, suggesting that GAGE expression is not associated with a specific genotype, but is linked to a specific phenotype. It has recently become evident that some tumours consist of a heterogeneous population of cells with a hierarchical organization, and that the capability of sustained tumour growth resides exclusively within a small proportion of cells that posses stem cell-like characteristics (Al-Hajj *et al*, 2003; Bapat *et al*, 2005; Ponti *et al*, 2005). Furthermore, it has been shown that a similar organization exists in some cancer cell lines (Kondo *et al*, 2004; Setoguchi *et al*, 2004; Ponti *et al*, 2005). The clonogenic nature of GAGE expression in cells of the genetically homogenous BrCa-MZ01 subclones suggests that expression of GAGE proteins is associated with a hierarchical distinct cell population. As we and others have shown that GAGE proteins are expressed in different types of stem cells (e.g. spermatogonia, oocytes, human mesenchymal stem cells (Cronwright *et al*, 2005) and haematopoietic stem cells (Guinn *et al*, 2005)), GAGE expression may define a population within the BrCa-MZ01 cell line that has the characteristics of cancer stem cells. A link between GAGE and self-renewal is further supported by the high frequency of GAGE-positive subclones (4/5) derived from the original BrCa-MZ01 cell line, which had only about 5% of GAGE-positive cells. Further studies will determine if GAGE proteins are markers of cancer stem cells and if the heterogeneous expression of GAGE proteins in tumours is a consequence of GAGE expression being turned off as the cells develop towards a more committed phenotype.

Using our mAbs, we also assessed the GAGE expression in normal tissues. As expected, high reactivity was seen in the germ cells of the testicular seminiferous tubuli, where spermatogonia and primary spermatocytes exhibited expression of GAGE, while the secondary spermatocytes were unstained. This suggests that GAGE expression is downregulated when primary spermatocytes undergo meiosis and become secondary spermatocytes. Interestingly, we also observed variations in the intensity of GAGE nuclear staining among spermatogonia. Several subtypes of spermatogonia representing different stages in early spermatogenesis have been identified (de Rooij, 1998), and differences in the intensity of GAGE nuclear staining may be associated with different spermatogonial subtypes. MAGE-A1 and NY-ESO-1 were also highly expressed in spermatogonia. However, we found that MAGE-A1 expression was strictly cytoplasmic, in contrast to reports by others that MAGE-A1 is also expressed in the nucleus of spermatogonia (Takahashi *et al*, 1995). In fact, MAGE-A1 was found to interact with the nuclear proteins SKIP and HDAC1 and thereby inhibit transcriptional activation mediated by Notch-IC (Laduron *et al*, 2004). The nuclear localization of GAGE proteins in spermatogonia suggests that GAGE may also be a regulator of germline gene expression.

GAGE proteins were also detected in oocytes of both resting and maturing follicles of the ovary, which is surprising since no other CT antigen has been identified in postfoetal oocytes, but also logical, since both oocytes and spermatogonia are derived from the foetal primordial germ cells. Only about 30% of oocytes of primordial resting follicles were positive and, based on the morphology, it was not possible to differentiate between the oocytes that exhibited GAGE staining and those that did not. The nature of this difference in GAGE expression is currently being investigated. Although present in foetal oogonia (Simpson *et al*, 2005), MAGE-A1 and NY-ESO-1 were not detected in oocytes. Thus, regulation of GAGE, MAGE-A1 and NY-ESO-1 gene expression in normal cells seems to depend on different mechanisms. Our observation that GAGE proteins are expressed in oocytes may not limit its use as a target for cancer-specific immunotherapy, since oocytes, similar to testicular germ cells, are recognized as immunoprivileged cells (Fenichel *et al*, 1995; Hutter and Dohr, 1998).

## Figures and Tables

**Table 1:**
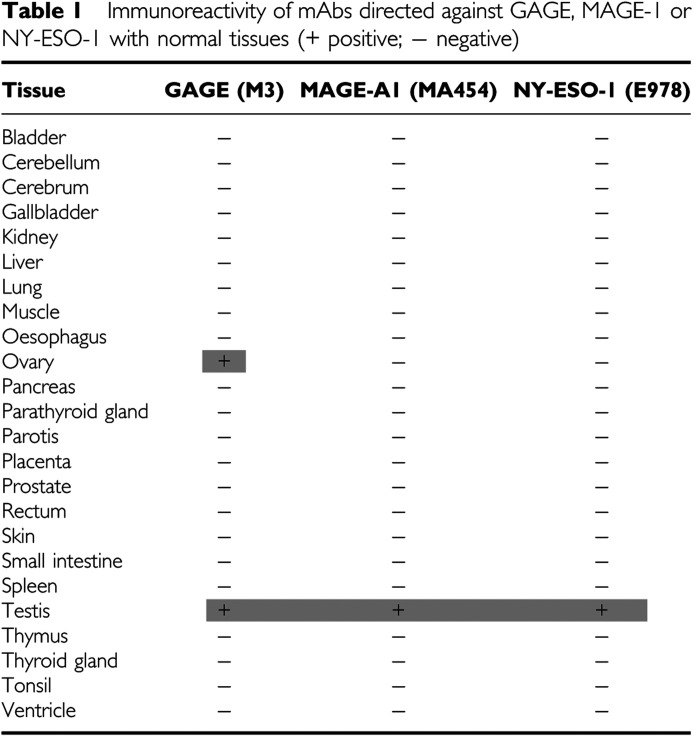
Immunoreactivity of mAbs directed against GAGE, MAGE-1 or NY-ESO-1 with normal tissues (+ positive; − negative)

**Table 2:**
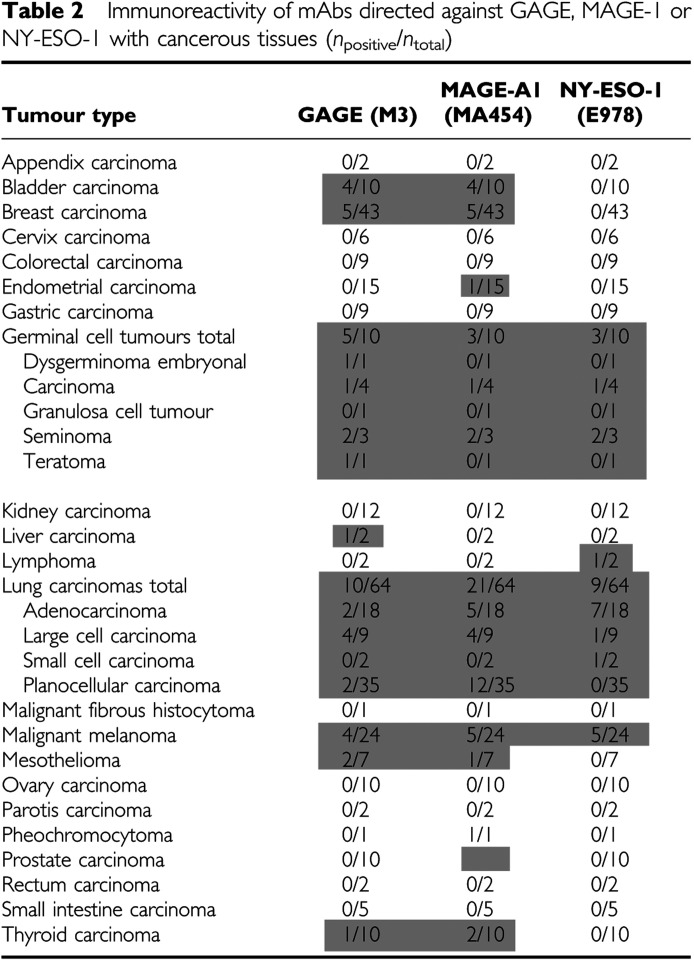
Immunoreactivity of mAbs directed against GAGE, MAGE-1 or NY-ESO-1 with cancerous tissues (*n*_positive_/*n*_total_)
